# Potential of food‐to‐food fortification with cowpea leaves and orange‐fleshed sweet potato, in combination with conventional fortification, to improve the cellular uptake of iron and zinc from ready‐to‐eat maize porridges

**DOI:** 10.1002/fsn3.1576

**Published:** 2020-05-18

**Authors:** Johanita Kruger

**Affiliations:** ^1^ Institute of Nutritional Sciences University of Hohenheim Stuttgart Germany; ^2^ Department of Consumer and Food Sciences and Institute of Food Nutrition and Well‐being University of Pretoria Hatfield Pretoria South Africa

**Keywords:** cowpea leaves, food‐to‐food fortification, iron, maize, orange‐fleshed sweet potato, zinc

## Abstract

An emerging tool in the fight against the high prevalence of micronutrient deficiencies in sub‐Saharan Africa is the production of nutritionally enhanced staple food products, through food‐to‐food fortification with micronutrient‐dense fruits and vegetables. This study investigated food‐to‐food fortification with cowpea leaves (CL) and orange‐fleshed sweet potato (OFSP) in combination with conventional micronutrient fortification and fermentation on the mineral and antinutrient contents and Caco‐2 cellular uptake of iron and zinc from ready‐to‐eat maize porridges. The amount of iron and zinc taken up from maize porridges (0.05 and 0.06 mg/100 g, db, respectively) was increased more after fortification with CL, compared to OFSP (0.32 and 0.23 mg/100 g, db versus. 0.11 and 0.04 mg/100 g, db, respectively). Despite the moderate cellular uptakes of iron and zinc from the CL fortified porridges (2.71% and 3.10%, respectively) compared to the OFSP fortified porridges (6.51% and 5.22%, respectively), the CL fortified porridges had much higher high iron and zinc contents (12.2–14.1 and 7.6–8.9 mg/100 g, db versus. 2.1–3.7 and 1.5–2.7 mg/100 g, db, respectively). This highlights the importance of increasing both the mineral content and bioavailability when fortifying a product. Even when a food product contains substantial antinutrients such as CL, if the mineral content and contents of bioavailability enhancers are high enough, the amounts of bioavailable iron and zinc can still be improved.

## Graphical abstract

This study investigated food‐to‐food fortification with cowpea leaves (CL) and orange‐fleshed sweet potato (OFSP) on the mineral nutritive value of ready‐to‐eat maize porridges. The amount of iron and zinc taken up from maize porridges by Caco‐2 cells was higher after fortification with CL, compared to OFSP. Despite the moderate cellular uptakes of minerals from the CL fortified porridges compared to the OFSP fortified porridges, the CL fortified porridges had much higher high iron and zinc contents. This highlights the importance of increasing both the mineral content and bioavailability when fortifying a product.

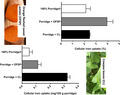

## 1 INTRODUCTION

**Figure 1:**
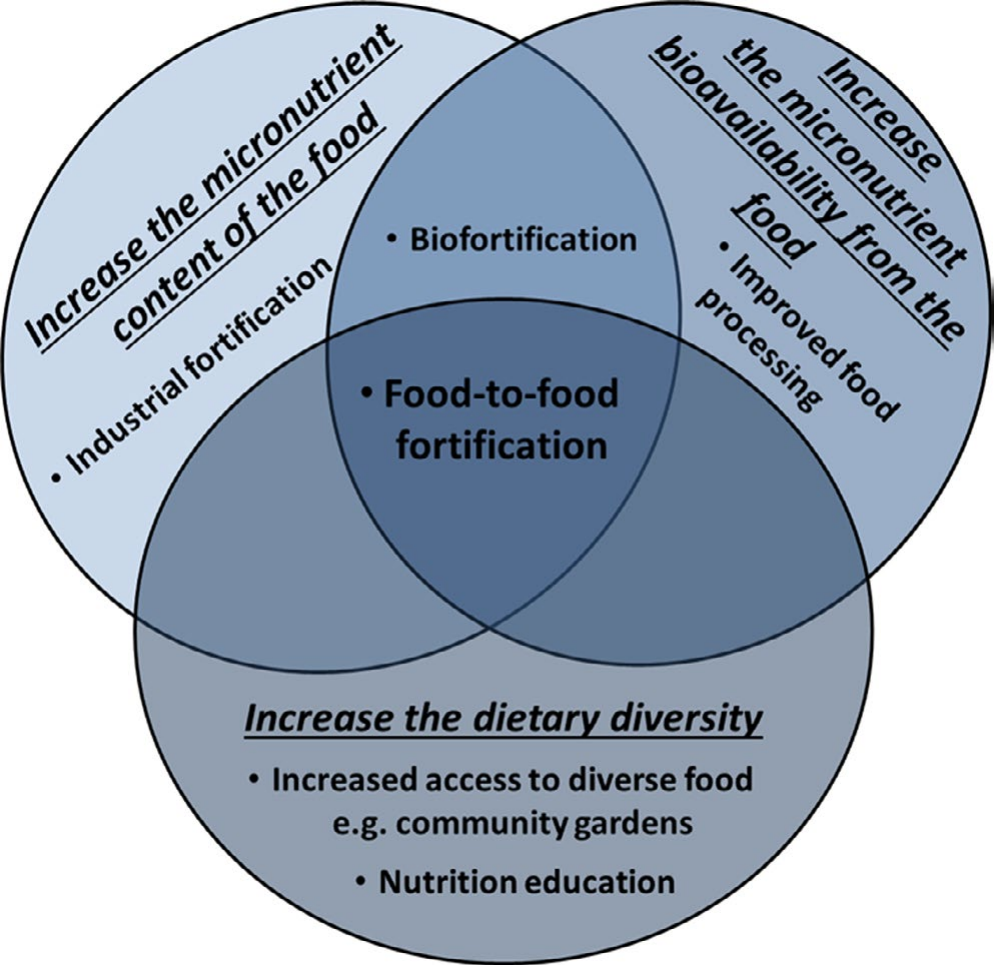
Main food‐based strategies to address micronutrient deficiencies caused by inadequate intake/absorption of nutrients due to the consumption of inadequate diets

While up to date data are scarce, the high prevalence of anemia and stunting suggests that iron and zinc deficiencies are still highly prevalent in sub‐Saharan Africa, often due to the consumption of monotonous cereal‐based diets and/or diets high in sugar/fat, but poor in micronutrients (Development Initiatives, 2018). Three main food‐based strategies are used to address nutrient deficiencies caused by chronic inadequate intake/absorption of micronutrients from these types of inadequate diets (Figure 1): increasing the micronutrient content of the food/diet, increasing the micronutrient bioavailability from the food/diet, and diversifying the diet to include more micronutrient‐dense foods.

An emerging tool, in the fight against the high prevalence of micronutrient deficiencies in sub‐Saharan Africa, is the nutritionally enhanced staple food products produced through food‐to‐food fortification with micronutrient‐dense fruit and vegetables. Food‐to‐food fortification not only increases the nutrient content of food products, but importantly, the nutrient‐dense fruit and vegetables used often also contain compounds such as organic acids, carotenoids, and vitamins, which act as nutrient bioavailability enhancers, especially of minerals (Sharma, 2003). This means all three main strategies to address chronic nutrient deficiencies are incorporated into food‐to‐food fortification (Figure 1).

As all current information suggests that iron, zinc, and vitamin A deficiencies are still the most prevalent forms of malnutrition throughout the world (Development Initiatives, 2018), food fortificants often contain high amounts of these micronutrients and bioavailability enhancers (e.g., organic acids or lipids), specific to each micronutrient.

Two important food fortificants identified are dark green leafy vegetables and orange‐fleshed sweet potato (OFSP). Dark green leafy vegetables are popular in addressing iron and zinc deficiencies, because, despite their high phytate and phenolic contents, they often contain very high levels iron and zinc, as well as bioavailability enhancers such as organic acids and β‐carotene (Uusiku et al., 2010). OFSP, often developed or improved through biofortification programs, is widely available throughout the developing world, and their potential in addressing vitamin A deficiencies has been widely documented (Laurie, Faber, Adebola, & Belete, 2015). The main goal of food‐to‐food fortification with OFSP is to increase the amounts of bioavailable provitamin A. It is however also important to evaluate the effect of this fortification on the iron and zinc nutritive value of the food product. OFSP, compared to staple grains, is lower in iron and zinc bioavailability inhibitors (phytate and phenolics) and high in enhancers (organic acids and β‐carotene) (Laurie et al., 2015). The contributions the food fortificants would make toward the energy content of the fortified food are also very important, especially in areas where hunger is prevalent. OFSP is considered a staple food and would provide more energy (1,734 kJ/100 g, db) compared to CL (1,187 kJ/100 g, db) (USDA, 2019).

Food‐to‐food fortification should, however, be seen as an additional food‐based approach, which should complement other strategies such as conventional fortification and improved food processing (Chadare et al., 2019). Conventional fortification of industrially milled wheat is mandatory in 26 African countries and of maize in nine, respectively, with an additional eight countries voluntarily fortifying more than 50% of their wheat/maize (FFI, 2019). The cereals are normally fortified with multi‐micronutrient fortificants, differing in composition, but in general containing at least iron, zinc, vitamin A, and folic acid. While elemental iron has traditionally been used as iron fortificant of cereal flours, sodium ferric ethylenediaminetetraacetate (NaFeEDTA) is becoming increasingly popular, as it has a higher bioavailability without the undesired sensory effects, which are observed with other high bioavailability fortificants, such as iron sulfate (Bothwell & Macphail, 2004; Sadighi, Nedjat, & Rostami, 2019). As for improved food processing; lactic acid bacteria fermentation of cereals has been found to improve both their nutrient contents (e.g., B vitamins) and bioavailability (e.g., minerals and protein) (Charalampopoulos, Wang, Pandiella, & Webb, 2002). Commercially fermented cereal products are also becoming more popular, as urbanization and changes in lifestyle decrease in‐home fermentation.

Conventional food fortification, however, still faces several issues, especially in developing countries (Chadare et al., 2019), and improved food processing methods are also limited in the extent to which nutrient content and bioavailability can be enhanced (Hotz & Gibson 2007). For these reasons, it is widely accepted that no single approach would rid the world of micronutrient deficiencies, but rather that a combined approaches should be followed. This study investigated food‐to‐food fortification with CL and OFSP in combination with conventional fortification and fermentation on the mineral and antinutrient contents and Caco‐2 cellular uptake of iron and zinc from a ready‐to‐eat maize porridge product.

## 2 MATERIALS AND METHODS

**Figure 2:**
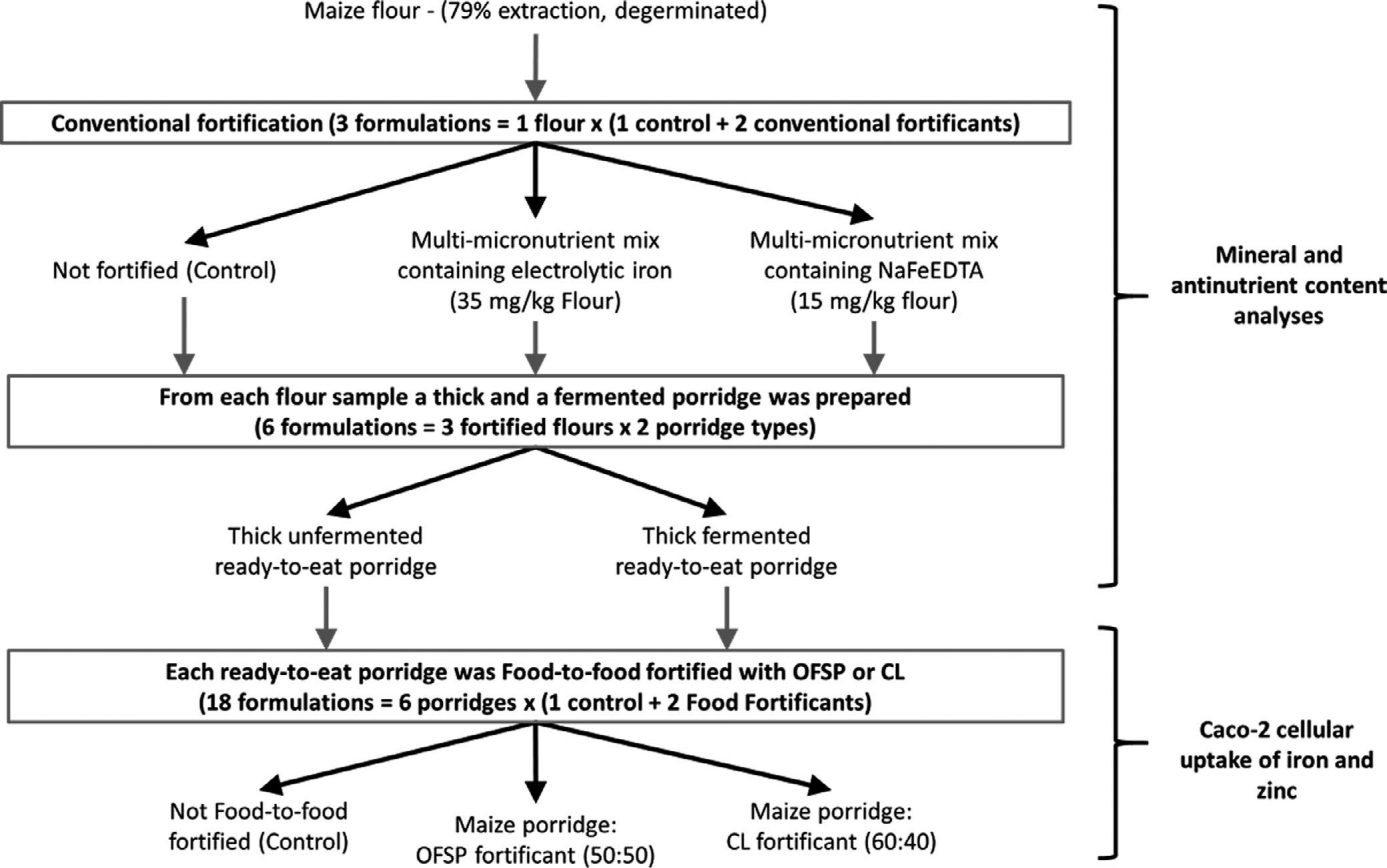
Experimental design

The experimental design is displayed in Figure 2.

### 2.1 Preparation of conventionally fortified maize flours

Refined maize flour was used to prepare the conventionally fortified maize flours (79% extraction, degerminated, kindly donated by Premier Mills). The two multi‐micronutrient fortification mixes used were prepared with either electrolytic iron or NaFeEDTA and formulated to ensure 35 and 15 mg iron/kg fortified flour, respectively (kindly donated by DSM) (Table 1). Due to the increased bioavailability of NaFeEDTA, the suggested fortification level is lower than that of electrolytic iron (WHO, 2009). The fortification mixes were each added to 10 kg of maize flour, and homogeneity was confirmed by analyzing representative subsamples (*n* = 4) for iron and zinc.

**Table 1:** Conventional multi‐micronutrient fortification (per kg) of refined maize flour

	Micronutrient fortificant	Micronutrient/ kg maize flour
Fortification base^b^	Vitamin A palmitate (activity: 75,000 µRE^b^/g)	2,085 µ RE
	Thiamine mononitrate (activity: 78% min) (mg)	2.19
	Riboflavin (mg)	1.69
	Nicotinamide/niacinamide (mg)	25.00
	Pyridoxine HCl (activity 81% min) (mg)	3.13
	Folic acid (90.5% min) (mg)	2.00
	Zinc oxide (activity: 80% min) (mg)	35.00

	Iron fortificant	Iron/ kg maize flour
Iron fortification	Fortification base with electrolytic iron (activity: 98% min) (mg)	35.00
	Fortification base with NaFeEDTA (activity: 13% min) (mg)	15.00

^a^ Fortification mix according to South African regulations (DOH, 2003).

^b^ Retinol equivalents (RE) = 1 µ retinol = 3.33 IU (International units) vitamin A.

### 2.2 Preparation of cowpea leaf and orange‐fleshed sweet potato powders

Young cowpea leaves (CL) (handpicked at the Ukulima Research Farm) and Bophelo variety orange‐fleshed sweet potato (OFSP) (kindly donated by the Agricultural Research Council, Roodeplaat, Vegetable and Ornamental Plants institute) were used to prepare the food fortificant powders (Kruger, Breynaert, Pieters, & Hermans, 2017). In short, the OFSP was rinsed with distilled water, boiled whole, with the skin on, cooled to room temperature, and cut into approximately 1.5 cm^2^ cubes. The cowpea leaves were thoroughly rinsed with distilled water (30 s), drained for 2 min, and cooked in the residual water, which was not discarded. After cooking, both the CL and OFSP were frozen at −20˚C, freeze‐dried, crushed, and passed through a 500‐µm screen to ensure homogenous fortificant powders, which were stored at −20˚C in airtight, opaque containers.

### 2.3 Preparation of fortified ready‐to‐eat porridges

The normal and fermented thick maize porridges were prepared according to Kruger (2016). In short, for the fermented maize flour, a starter culture was prepared by mixing unfortified maize flour and distilled water (1:2 w/w) which was incubated at 25˚C until the pH was <4 (36–48 hr). The fermented flour samples were prepared by mixing the respective conventionally fortified maize flours, distilled water, and the starter culture (10:20:1 w/w). The flour slurry mix was then incubated as described (24–36 hr). The fermented flour slurry was frozen at −20˚C, freeze‐dried, crushed, and passed through a 500‐µm screen to ensure homogenous flour.

To prepare the ready‐to‐eat (precooked) porridge, distilled water was added to fermented or unfermented maize flour (10:1 w/w). The mixtures were heated to boiling and maintained, with constant stirring, for 15 min. The porridges were left to cool at room temperature and then frozen at −20˚C before being freeze‐dried.

To prepare the ready‐to‐eat, food‐to‐food fortified maize porridges, the precooked and dried porridge powders were mixed with the precooked and dried CL and OFSP fortificant powders at a 60:40 and 50:50 ratio, respectively. This is in line with meal combinations including both a porridge and OFSP or CL relish, respectively (Kruger et al., 2017) and slightly higher than 30% fortification of pearl millet porridge with moringa leaf powder. Also, a recent review found that food‐to‐food fortificants are used at substitution levels of between 1% and 50% (Chadare et al., 2019). It is however important to state that as this is the first research on using OFSP and CL as food fortificants of maize porridges, the sensory acceptability of these levels of fortification, however, still needs to be evaluated.

### 2.4 Analyses

All individual porridge and fortificant powders were analyzed for mineral and antinutrient contents. The iron and zinc cellular uptake was measured from the fortificant powders and the ready‐to‐eat, food‐to‐food fortified porridges (Figure 2).

#### 2.4.1 Antinutrient contents

Phytate content was determined using an indirect quantitative anion exchange method (Fruhbeck, Alonso, Marzo, & Santidrián, 1995). Total phenolic content was determined using a modified Folin–Ciocalteu method (Kaluza, McGrath, Roberts, & Schroeder, 1980). Tannin content was determined using a modified vanillin‐HCl assay (Price et al., 1978). The modification in both the total phenolic and tannin assays was that reagent blanks were included to correct for the color of the fortificant powder extracts.

#### 2.4.2 Mineral contents

The total mineral contents (Al, Fe, Zn, Mg, Ca, and P) were analyzed using ion coupled plasma–optical emission spectrometry (ICP‐OES), after acid digestion (Kruger, Mongwaketse, Faber, Hoeven, & Smuts, 2015).

#### 2.4.3 Caco‐2 cellular uptake of iron and zinc

The cellular uptake of iron and zinc was measured using a Caco‐2 cell uptake model, as described by (Kruger et al., 2012). For the in vitro digestion, crystallized, lyophilized, and essentially salt‐free pepsin (porcine, 4,200 U/mg), pancreatin, (P‐1750), and bile extract (B‐8631) (Sigma) were used.

Caco‐2 cells were kindly donated by the Department of Pharmacology, North‐West University and cultured in Dulbecco's modified Eagle medium (DMEM) with glucose, Earle's salts, and L‐glutamine (Hyclone) containing 10% fetal bovine serum, which was not heat‐treated (Highveld Biological), and 1% penicillin/streptomycin (Hyclone). All experiments were conducted between passages 12–20.

Iron and zinc radio isotope labeling was achieved by mixing digested porridges in equal parts with the same medium used to culture the cells, except with 2% bovine serum. ^59^Fe and ^65^Zn (Separations Scientific) in the form of ^59^FeSO4 and ^65^ZnCl_2_ were used. The samples were left overnight for the isotopes to exchange with the intrinsic iron and zinc.

The isotope labeled samples were applied to the Caco‐2 cells, which were incubated for 6 hr, after which the porridge: Medium mix and cells were collected separately and counted in a Wizard2 2,470 automatic gamma counter (Wallac, Perkin Elmer). Results are presented as the percentage of radioactivity in the cells, relative to the total activity in the well (cellular uptake [%]), and as the amount of iron or zinc taken up by the Caco‐2 cells, cellular uptake (mg/100 g porridge) = iron/zinc content (mg/100 g) x cellular uptake (%).

### 2.5 Calculations

#### 2.5.1 Contribution to recommended dietary allowance (RDA) of iron and zinc

The contribution a porridge portion could make toward the recommended dietary allowance (RDA) of iron and zinc of healthy women of childbearing age was calculated based on the following: A 250 g porridge portion was chosen based on the study by Nesamvuni, Potgieter, and Steyn (2001) who found that women on average consumed approximately 850 g of a staple maize porridge per day. This portion size was based on three meals and considered average which would not overestimate the nutrient contribution. The calculations were based on a stiff porridge formulation (25% solids). The calculated iron and zinc contents were compared to the requirements of healthy females of childbearing age for iron (18 mg/day) and zinc (8 mg/day) (IOM, 2001).

#### 2.5.2 Phytate:mineral molar ratios

Molar ratios of Phytate:Fe, Phytate:Zn, and PhytatexCa:Zn were calculated as follows:

$$\text{Molar ratio}\;=\;\left(\text{mole phytate}\right)\text{/(mole mineral)}.$$

### 2.6 Statistical analyses

All statistical analyses were done using Statistica 12 (StatSoft). Normality of data and equality of variance were assessed using the Shapiro−Wilk and Levene tests, respectively. To analyze differences between groups (phytate, total phenolic, and tannin contents [*n* = 4], Caco‐2 uptake [*n* ≥ 9]), one‐way analysis of variance (ANOVA) and main‐effects ANOVA were used. Fisher's LSD post hoc test was used to determine differences between specific means at a confidence level of 95% (*p* ≤ .05).

## 3 RESULTS AND DISCUSSION

### 3.1 Effects of food‐to‐food fortification in combination with conventional multi‐micronutrient fortification and fermentation on the mineral and antinutrient contents of ready‐to‐eat maize porridges

The mineral contents of the OFSP, CL, and maize flour used (Table 2) were similar to those reported by others. According to the USDA (USDA 2019), the average mineral contents (db) for white, refined, and degerminated maize flour are 1.2 mg/100 g for iron, 0.74 mg/100 g for zinc, 2.7 mg/100 g for calcium, 91 mg/100 g for phosphorus, and 35 mg/100 g for magnesium. Laurie et al. (2015), in a review of various OFSP varieties, reported ranges (db) of 3.3–4.4 mg/100 g for iron, 2.1–2.8 mg/100 g for zinc, 220–246 mg/100 g for calcium, 138–205 mg/100 g for phosphorus, and 108–165 mg/100 g for magnesium. While the iron content of the CL was similar to that found by Goncalves et al. (2016) (16–77 mg/100 g, db), the zinc and calcium contents were higher than the reported ranges of 0.3–12.9 mg/100 g (db) for zinc and 10–805 mg/100 g (db) for calcium. Mineral contents of green leafy vegetables vary substantially due to varietal and environmental differences and contamination of especially ground‐level growing leaves such as CL (Gonçalves et al., 2016). The high aluminum content of the CL powder (11.0 mg/100 g, db) could be an indication of soil contamination. Despite thorough cleaning, green leafy vegetables are often still contaminated with soil/dust adhering to the leaves, and soaking in a dilute acid is often necessary to remove all of these contaminants (van Jaarsveld et al., 2014). The cleaning protocol in this study, however, was similar to that which would be used by small business enterprises to produce a fortificant powder.

**Table 2:** Mineral, phytate, total phenolic, and tannin contents of unfortified maize flour, cowpea leaf (CL), and orange‐fleshed sweet potato (OFSP) powders

	Iron	Zinc	Calcium	Phosphorus	Magnesium	Aluminum	Phytate	Tannins	Total phenolics
	(mg/100 g, db)							(mg CE/100 g, db)	
Maize flour†	0.8 ± 0.1^a^	0.8 ± 0.1^a^	4 ± 0^a^	118 ± 3^a^	38 ± 1^a^	0.7 ± 0.5^a^	474 ± 36^b^	ND	138 ± 11^a^
OFSP	3.3 ± 0.3^b^	2.2 ± 0.3^b^	219 ± 10^b^	399 ± 1^b^	148 ± 2^b^	3.2 ± 1.0^a^	203 ± 20^a^	ND	869 ± 34^b^
CL	29.3 ± 1.0^c^	17.6 ± 0.7^c^	2,083 ± 6^c^	1,723 ± 28^c^	667 ± 7^c^	11.0 ± 1.9^b^	631 ± 12^c^	851 ± 20	5,187 ± 70^c^

Note

Values are displayed as means ± *SD* (*n* = 4).

Abbreviations: CE, catechinequivalents; db, dry basis; ND, not detected.

^†^ From Kruger (2016).

^abc^Means in the same row with different superscripts, differ significantly (p ≤ .05).

The CL powder, like other dried dark green leaves (Gonçalves et al., 2016; van Jaarsveld et al., 2014), was concentrated in nutrients and antinutrients. The powder contained considerably higher amounts of phytate, tannins, total phenolics, and minerals including iron and zinc, than the maize flour (Table 2), even after the maize flour was conventionally fortified with iron and zinc (Table 3). While the OFSP also contained more minerals than the unfortified maize flour (Table 2), the level of iron was similar and zinc somewhat lower, after the maize flour was conventionally fortified (Table 3). The OFSP, when compared to the maize flour, also contained no measurable amounts of tannins, only half the amount of phytate, but sixfold more total phenolics (Table 2).

**Table 3:** Effect of multinutrient fortification and food‐to‐food fortification with cowpea leaf (CL) and orange‐fleshed sweet potato (OFSP) powders on the iron and zinc contents of maize porridge (MP) and the potential contribution a 250 g porridge portion (as consumed) can make toward the recommended dietary allowances (RDA) of healthy women

	Fe (mg/100 g, db)			Zn (mg/100 g, db)		
	100% MP (100:0)^a^	MP: OFSP (50:50)^b^	MP: CL (60:40)^b^	100% MP (100:0)^a^	MP: OFSP (50:50) ^b^	MP: CL (60:40) ^b^
Unfortified	0.8 ± 0.1 [3%]	2.1 ± 0.3 [7%]	12.2 ± 1.5 [42%]	0.8 ± 0.1 [6%]	1.5 ± 0.2 [12%]	7.6 ± 1.0 [59%]
Electrolytic Fe fortification	4.0 ± 0.6 [14%]	3.7 ± 0.6 [13%]	14.1 ± 2.1 [49%]	2.8 ± 0.5 [22%]	2.6 ± 0.4 [20%]	8.7 ± 1.6 [68%]
NaFeEDTA fortification	2.2 ± 0.7 [8%]	2.8 ± 0.9 [10%]	13.1 ± 4.2 [45%]	3.1 ± 0.1 [24%]	2.7 ± 0.1 [21%]	8.9 ± 0.3 [70%]

Note

Values are displayed as means ± *SD* (*n* = 4). In square brackets, the percentage contribution a 250 g portion of porridge (as consumed, 25% solids) can make toward the RDA of healthy females of childbearing age for iron (18 mg/ day) and zinc (8 mg/ day) (IOM, 2001).

^a^ From Kruger (2016).

^b^ Values calculated on a formulation of 50:50 MP: OFSP powder and 60:40 MP: CL powder.

Fortifying the maize porridges with the CL, and to a lesser extent the OFSP, improved the contribution a 250 g porridge portion could make toward the recommended dietary allowance (RDA) of iron and zinc of healthy women of childbearing age (Table 3). Addition of the CL powder increased the iron and zinc contents of the unfortified and conventionally fortified porridges 14‐ and fivefold, and nine‐ and threefold, respectively. A 250 g portion of the CL fortified maize porridge could provide 40%–50% and 60%–70% of the iron and zinc RDA of healthy women of childbearing age, respectively, compared to 8%–14% and 22%–24% from the conventionally fortified maize porridges. Addition of the OFSP powder more than doubled the iron and zinc contents of the unfortified maize porridge, but had no effect on the iron content of the fortified maize porridges and slightly reduced (by approx. 10%) the zinc content of the conventionally fortified maize porridges. A 250 g portion of the OFSP fortified maize porridges could provide 7%–13% and 12%–21% of the iron and zinc RDA of healthy women, respectively. This indicates that the conventional fortification and food‐to‐food fortification with OFSP have similar capacity to increase the iron and zinc intake from the maize porridges. The critical question however is, whether the added iron and zinc, either from the conventional or food fortificants, are bioavailable.

### 3.2 Effects of food‐to‐food fortification in combination with conventional multinutrient fortification and fermentation on the cellular uptake (%) of iron and zinc from ready‐to‐eat maize porridges

The cellular uptake of iron and zinc from the OFSP fortified porridges, which ranged from 3.18% to 4.94% and 2.85% to 3.73%, respectively, was lower than that from the OFSP powder alone (6.51% and 5.22%, respectively) (Table 4). Fermentation only enhanced the cellular uptake (%) of iron and zinc from the OFSP and electrolytic iron fortified porridge. Simultaneous fortification with OFSP and the NaFeEDTA fortification mix, however, resulted in higher cellular uptake (%) of both iron (4.92%–4.94%) and zinc (3.63%–3.73%), compared to the other porridges (3.18%–4.01% and 2.85%–3.09%, respectively). NaFeEDTA fortification of maize porridges has previously also been found to increase the cellular uptake of both iron and zinc and was explained by the dissociation of NaFeEDTA complexes due to pH changes during the digestion, after which Zn‐EDTA complexes might form, also increasing the cellular uptake (%) of zinc (Kruger, 2016).

**Table 4:** Cellular uptake (%) of iron and zinc from thick and fermented maize porridges, fortified with cowpea leaf (CL), orange‐fleshed sweet potato (OFSP), and/or a conventional multinutrient fortification mix

	Orange‐fleshed sweet potato		Cowpea leaf	
	Thick porridge	Fermented thick porridge	Thick porridge	Fermented thick porridge
	Caco‐2 cellular uptake (%) of iron			
Food‐to‐food fortificant alone	6.51 ± 0.96^d^		2.71 ± 0.34^a^	
Unfortified maize flour	3.46 ± 0.20^a^	3.18 ± 0.09^a^	2.29 ± 0.23^a^	2.50 ± 0.17^a^
Electrolytic Fe mix fortified maize flour	3.55 ± 0.98^a^	4.01 ± 1.44^b^	2.46 ± 0.18^a^	2.55 ± 0.30^a^
NaFeEDTA mix fortified maize flour	4.92 ± 0.41^c^	4.94 ± 0.22^c^	2.32 ± 0.32^a^	2.57 ± 0.30^a^
	Caco‐2 cellular uptake (%) of zinc			
Food‐to‐food fortificant alone	5.22 ± 0.49^d^		3.10 ± 0.45^b^	
Unfortified maize flour	2.91 ± 0.28^a^	2.94 ± 0.68^a^	3.05 ± 0.4^ab^	2.85 ± 0.37^ab^
Electrolytic Fe mix fortified maize flour	2.85 ± 0.67^a^	3.09 ± 0.69^b^	2.86 ± 0.38^ab^	2.69 ± 0.38^a^
NaFeEDTA mix fortified maize flour	3.63 ± 0.49^c^	3.73 ± 0.5^c^	2.91 ± 0.51^ab^	3.05 ± 0.17^ab^

Note

Values are displayed as means ± *SD* (*n* ≥ 9). ^abc^Means of iron or zinc uptakes of each food fortificant, with different superscripts, differ significantly (*p* ≤ .05).

**Figure 3:**
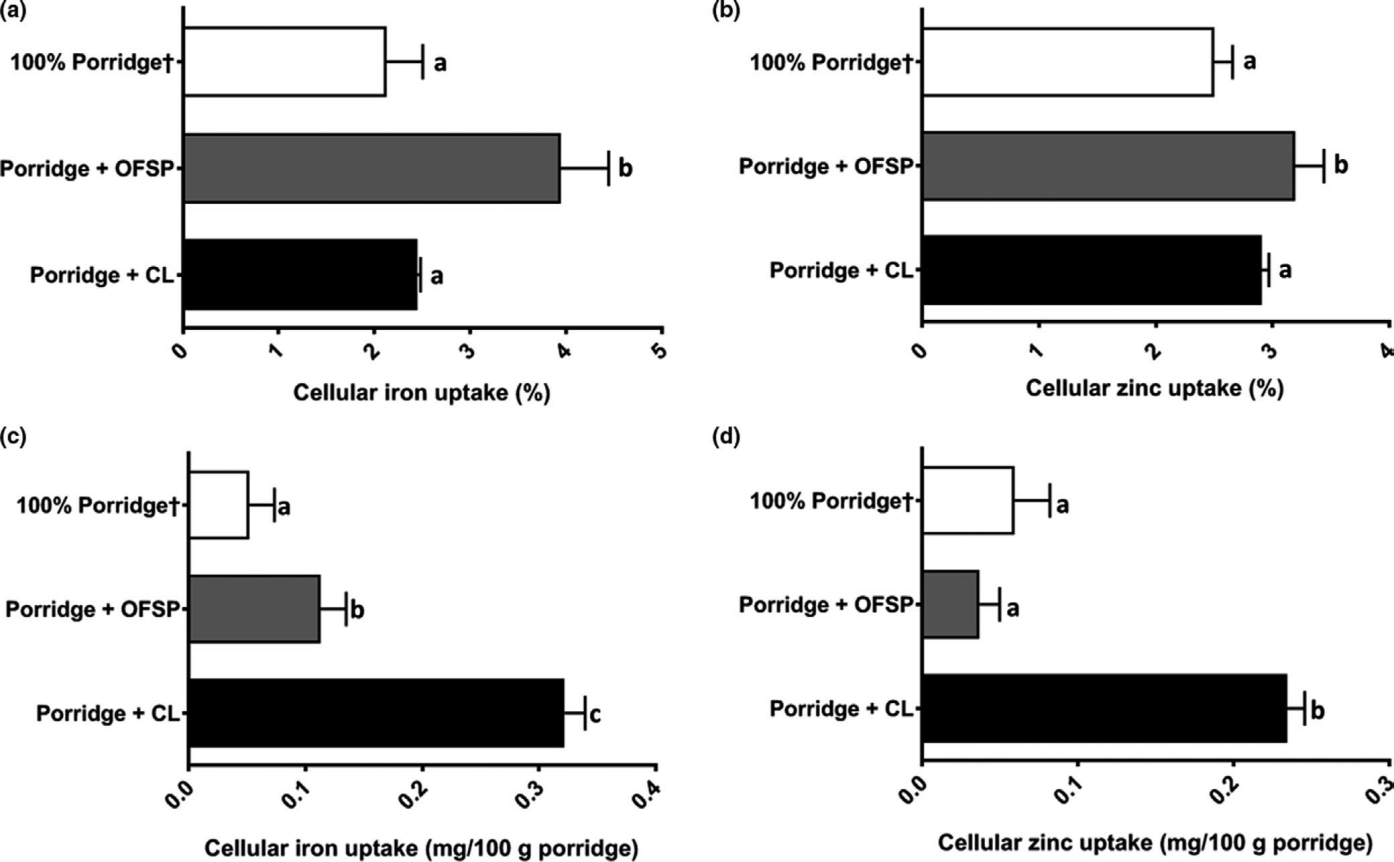
Main effects of food‐to‐food fortification with orange‐fleshed sweet potato (OFSP) and cowpea leaves (CL)on the Caco‐2 iron and zinc cellular uptake (%) and the amount of iron and zinc taken up by the Caco‐2 cells (mg/100 g flour) from ready‐to‐eat maize porridges. ^abc^Columns within each figure with different letters (*n* = 6) differ significantly (*p* ≤ .05), ^†^From Kruger (2016)

A multivariate ANOVA of the cellular uptake (%) comparing the 100% maize and food‐to‐food fortified porridges (including all fermented and commercially fortified porridges) revealed that OFSP fortification resulted in overall increased cellular uptake of iron and, to a lesser extent, zinc (Figure 3a,b). This was despite the much higher total phenolic content of the OFSP, compared to the maize flour (Table 2), which was expected to inhibit the cellular uptake of iron and zinc. The extent of iron and zinc bioavailability inhibition by phenolics, however, very much depends on the phenolic profile of the food (Brune, Hallberg, & Skånberg, 1991). The phenolic profile of the OFSP might not have resulted in high iron and zinc uptake inhibition. The higher cellular uptake could also have been because the phytate content of the OFSP was less than half of the maize flour and OFSP also contains various enhancers of iron and zinc bioavailability such as ascorbic acid and β‐carotene. Both ascorbic acid (Teucher, Olivares, & Cori, 2004) and β‐carotene (García‐Casal et al., 1998) have been found to increase the bioavailability of iron, while the effect on zinc bioavailability is less clear. The ascorbic acid content of various sweet potatoes has been found to vary between 66 to 87 mg/100 g, with no relationship between the sweet potato color and ascorbic acid content (Grace et al., 2014). Bophelo OFSP (variety used in this study) has been found to contain approximately 34 mg β‐carotene/100 g (db), which is lower than other OFSP varieties, with reported contents of up to 82 mg/100 g (db) (Laurie et al., 2015). However, at a provitamin A conversion rate of 12, a 250 g portion of the Bophelo OFSP fortified porridges (estimated 60% retention of β‐carotene after processing and storage) could provide 75% of the vitamin A RDA of women of childbearing age (700 µg RAE/day) (IOM, 2001).

The cellular uptake (%) of iron and zinc from maize porridges fortified with CL and the CL powder alone ranged from 2.29% to 2.57% and 2.69 to 3.10%, respectively (Table 4). When fortified with CL, neither conventional fortification nor fermentation of the porridges had any effect on the cellular uptake of iron or zinc, probably due to the high concentrations of minerals and antinutrients of the CL; even at a fortification ratio of 60:40 maize: CL, the vast majority of the iron and zinc (64%–94%), tannins (100%), and total phenolics (96%), the ready‐to‐eat porridges were still from the CL (Tables 1 and 3).

Interestingly, despite the high antinutrient contents of CL (Table 1), a multivariate ANOVA of the cellular uptake (%), comparing the 100% maize and food‐to‐food fortified porridges (including all fermented and commercially fortified porridges), did not show a decrease in the cellular uptake (%) of iron and zinc (Figure 3a,b). Despite the 25% higher phytate content of the CL, the phytate:Fe ratios of the CL fortified porridges (4–7) were, in general, much lower than that of the porridges without CL (4–57) (Table S1). This was due to the four‐ to 15‐fold higher iron contents of the CL fortified porridges, compared to the 100% maize porridges. All phytate:Fe ratios, however, were higher than the critical value of 1, above which iron bioavailability has been found to be seriously impaired (Hunt, 2003). Fortification of maize flour with CL, which had three‐ to 10‐fold higher zinc content than the maize porridges, decreased the phytate:Zn ratios of the maize porridges from 6–22 to 4–7, well below the critical value of 15 (Hunt, 2003). With such a reduction in phytate:zinc molar ratios, an increase in zinc cellular uptake (%) was expected, but not observed. Possibly due to the high calcium content of CL, the low phytatexCa:Zn ratios of the 100% maize porridges (0–6) were increased 25‐ to 83‐fold upon fortification with the CL. Another factor which could have mitigated the adverse effects of the mineral bioavailability inhibitors from the CL on the uptake of iron and zinc was the high levels of vitamins and other iron bioavailability enhancers in CL (Uusiku et al., 2010; Schönfeldt & Pretorius, 2011; van Jaarsveld et al., 2014). Like OFSP, CL is also high in β‐carotene (40 mg/100 g) and ascorbic acid (51 mg/100 g) (van Jaarsveld et al., 2014). Lastly, the high contents of divalent minerals (sum of Fe, Zn, Ca, Mg contents) in the CL versus. the maize flour (18‐ to fivefold higher) (Table 2) could also have diluted the inhibitory effects of the antinutrients (tannins and other phenolic compounds) on iron and zinc cellular uptake (Table 4).

### 3.3 Effects of food‐to‐food fortification in combination with conventional multinutrient fortification and fermentation on the amount of iron and zinc taken up by the Caco‐2 cells (mg/100 g porridge) from ready‐to‐eat maize porridges.

The percentage of cellular uptake (%) does not take into account the substantial variation in the iron and zinc contents between the maize, OFSP, CL, and respective fortified porridges (Table 1). For this reason, the amounts of iron and zinc taken up by Caco‐2 cells [Cellular uptake (mg/100 g porridge) = iron/zinc content (mg/100 g) x cellular uptake (%)] are also presented (Table 5). This makes it possible to compare the sum of the effects of the cellular uptake inhibitors and enhancers and the variation in the total iron and zinc contents between the different porridges (Kruger et al., 2015).

**Table 5:** Amount of iron and zinc taken up by Caco‐2 cells (mg/100 g db) from thick and fermented maize porridges, fortified with cowpea leaf (CL), orange‐fleshed sweet potato (OFSP), and/or a conventional multinutrient fortification mix

	Orange‐fleshed sweet potato		Cowpea leaf	
	Thick porridge	Fermented porridge	Thick porridge	Fermented porridge
	Caco‐2 cellular uptake (mg/100 g porridge) of iron			
Food‐to‐food fortificant alone	0.22 ± 0.03^b^		0.80 ± 0.10^c^	
Unfortified maize flour	0.07 ± 0.00^a^	0.07 ± 0.01^a^	0.28 ± 0.03^a^	0.30 ± 0.02^a^
Electrolytic Fe mix fortified maize flour	0.11 ± 0.01^ab^	0.15 ± 0.05^b^	0.35 ± 0.03^b^	0.34 ± 0.04^ab^
NaFeEDTA mix fortified maize flour	0.14 ± 0.01^b^	0.14 ± 0.01^b^	0.30 ± 0.04^a^	0.36 ± 0.04^b^
	Caco‐2 cellular uptake (mg/100 g porridge) of zinc			
Food‐to‐food fortificant alone	0.12 ± 0.01^c^		0.55 ± 0.08^b^	
Unfortified maize flour	0.01 ± 0.00^a^	0.01 ± 0.00^a^	0.22 ± 0.03^a^	0.21 ± 0.03^a^
Electrolytic Fe mix fortified maize flour	0.04 ± 0.01^b^	0.04 ± 0.01^b^	0.24 ± 0.03^a^	0.23 ± 0.03^a^
NaFeEDTA mix fortified maize flour	0.06 ± 0.01^b^	0.06 ± 0.01^b^	0.25 ± 0.04^a^	0.26 ± 0.01^a^

Note

Values are displayed as means ± *SD* (*n* ≥ 9). ^abc^Means of iron or zinc uptakes of each food fortificant, with different superscripts, differ significantly (*p* ≤ .05).

Even though the cellular uptake (%) of iron and zinc from the OFSP fortified porridges was higher than that of the CL porridges, larger amounts of iron and zinc were taken up by the Caco‐2 cells from the CL fortified porridges (Figure 3a,c). This means the CL fortified porridges have the potential to improve the iron and zinc absorption by the consumer to a greater extent than the OFSP, due to a combination of moderate cellular uptake (%) of the iron and zinc, with very much higher iron and zinc contents. The uptakes of iron and zinc from the CL fortified porridges were 0.28 to 0.36 mg/100 g and 0.21 to 0.26 mg/100 g, respectively, compared to the OFSP fortified porridges at 0.07 to 0.15 mg/100 g and 0.01 to 0.06 mg/100 g, respectively (Table 5). After fortification with CL, neither conventional fortification nor fermentation had any substantial effect on the amount of iron and zinc taken up by the Caco‐2 cells, as was also observed with the percentage of cellular uptake (Table 4). Conventional fortification in combination with OFSP fortification, however, resulted in increased amounts of iron and zinc being taken up by the Caco‐2 cells. This is because the iron and zinc from the conventional fortificant represented a substantial percentage of the total iron and zinc in these porridges, approximately 33 to 76% and 67 to 80%, respectively.

The overall effect of the fortification with OFSP and CL on the amount of iron and zinc taken up by the Caco‐2 cells (mg/100 g) compared to the 100% maize porridges is displayed in Figure 3 (c,d). Despite increased percentage of cellular uptake of both iron and zinc when the maize flour was fortified with OFSP, only the amount of iron taken up by the cells was increased. This was due to the lower zinc content of the OFSP, compared to the conventionally fortified maize porridges (Table 3). In contrast, CL fortification resulted in substantial increases the amounts of both iron and zinc taken up from the porridges (mg/100 g). This was due to the high contents of divalent minerals (including iron and zinc) and bioavailability enhancers such as β‐carotene and ascorbic acid in CL. This agrees with a recent study where it was found that a monotonous but micronutrient‐rich diet of a staple gain and dark green leafy vegetables (high in iron, β‐carotene, and antinutrients) was advantageous over a diet consisting mainly of a staple grain, in terms of iron status (Stuetz et al., 2019).

## 4 CONCLUSIONS

Firstly, this work highlights the importance that a food fortificant should increase both the mineral content and bioavailability, in order to improve the mineral absorption from a product. Even when a food product contains substantial antinutrients such as CL, if the mineral content and contents of bioavailability enhancers are high enough, the mineral bioavailability can still be improved. On the other hand, food fortificants with lower antinutrient contents and high contents of bioavailability enhancers, like OFSP, increase the percentage of cellular uptake of the minerals more. This could make these types of food fortificants good candidates to be combined with conventional fortification or food fortificants such as CL, which would increase the iron and zinc content. These findings however would need to be confirmed with human bioavailability studies.

## CONFLICT OF INTEREST

The author declares that she does not have any conflict of interest.

## Supporting information

Table S1
