# Supplementary material for: Potential of food‐to‐food fortification with cowpea leaves and orange‐fleshed sweet potato, in combination with conventional fortification, to improve the cellular uptake of iron and zinc from ready‐to‐eat maize porridges
Source: Food Sci Nutr. 2020 May 18;8(7):3190–9. doi: 10.1002/fsn3.1576 (PMC7382155; doi:10.1002/fsn3.1576)
Supplement: Supplementary file 1 — Table S1 [file FSN3-8-3190-s001.docx]

**Supplementary tables:**

**Supplementatry Table 1:** Phytate:iron, phytate:Zn and phytatexcalcium:zinc ratios of thick and fermented maize porridges, food-to-food fortified with cowpea leaf (CL) and orange-fleshed sweet potato (OFSP) powders with or without conventional multi-nutrient fortification

|  | Phytate:Fe ratio | | | Phytate:Zn ratio | | | PhytatexCa:Zn ratio | | |
| --- | --- | --- | --- | --- | --- | --- | --- | --- | --- |
|  | 100% Porridge | Porridge + CL | Porridge + OFSP | 100% Porridge | Porridge + CL | Porridge + OFSP | 100% Porridge | Porridge + CL | Porridge + OFSP |
| **Unfermented unfortified** | 57 | 7 | 22 | 57 | 7 | 22 | 6 | 147 | 61 |
| **Unfermented Electrolytic fortified** | 17 | 6 | 13 | 17 | 6 | 13 | 3 | 127 | 37 |
| **Unfermented NaFeEDTA fortified** | 15 | 6 | 13 | 15 | 6 | 13 | 2 | 125 | 35 |
| **Fermented unfortified** | 24 | 5 | 13 | 24 | 5 | 13 | 2 | 102 | 36 |
| **Fermented Electrolytic fortified** | 6 | 4 | 7 | 6 | 4 | 7 | 1 | 83 | 20 |
| **Fermented NaFeEDTA fortified** | 4 | 4 | 6 | 4 | 4 | 6 | 0 | 75 | 17 |
